# ‘Stories of Loss’—Designing and Evaluating a Patient‐Led Perinatal Bereavement Programme for Medical Students

**DOI:** 10.1111/tct.70146

**Published:** 2025-07-16

**Authors:** Clare Kennedy, Alison Lynch, Nina Doyle, Susanne Brodigan, Laura Guild, Mary F. Higgins

**Affiliations:** ^1^ UCD Perinatal Research Centre, National Maternity Hospital University College Dublin Dublin Ireland; ^2^ Leanbh Mo Chroí Ireland (LMC) Dublin Ireland; ^3^ Féileacáin Skibbereen Ireland; ^4^ Ectopic Pregnancy Dublin Ireland

**Keywords:** patient storytelling, patient voice, patient‐led education, patient‐teacher, perinatal bereavement, pregnancy loss

## Abstract

**Background:**

Providing bereavement support to parents who have experienced a perinatal loss requires knowledge, empathy and sensitivity. Undergraduate opportunities to learn directly from parents with personal experience remain limited. This study assessed medical students' responses to parent‐led stories on perinatal loss, evaluating their self‐reported changes in knowledge, skills and self‐awareness.

**Approach:**

Final‐year medical students in University College Dublin participated in a new educational initiative featuring parent‐educators from three perinatal loss advocacy groups. Each session focused on the parents' personal experiences of pregnancy loss and was designed collaboratively with the authors and independently led by the parents.

**Evaluation:**

Evaluation used a validated preintervention and postintervention questionnaire—the Perinatal Bereavement Care Confidence Scale (PBCCS). This measures self‐reported knowledge, support skills and self‐awareness in providing bereavement care. Students also provided free‐text comments on confidence promoters, inhibitors and suggestions for improvement. Statistically significant improvements were observed across all three domains of the PBCCS following the educational sessions. Knowledge scores increased 28.2%, from 2.80 to 3.59 (*p* < 0.01; Cohen's *d* = 0.39). Skills rose 43.1%, from 2.16 to 3.09 (*p* < 0.001; *d* = 0.65). Self‐awareness increased 21.6%, from 3.29 to 4.00 (*p* < 0.001). Thematic content analysis of free‐text responses revealed a lack of experience and fear of ‘saying the wrong thing’ as confidence inhibitors. Postintervention responses highlighted the value of hearing from the bereaved parents, with students calling for continued parent‐educator sessions.

**Implications:**

This pilot educational programme highlights the value of in‐person, parent‐led education and suggests that integrating the lived experience can better prepare students to provide bereavement care.

## 1 Background

The spectrum of perinatal loss encompasses the loss of a pregnancy at any stage. The impact of this loss on a person, their partner and their families can cause significant stress and emotional turmoil. The experience of grief is individual but can have lifelong psychological consequences [1]. Parents highlight the importance of sensitivity in clinical discussions; harm can be caused by poor communication and lack of sensitivity [2, 3].

Supporting parents who have experienced perinatal loss can be a challenging part of providing care [4, 5]. The National Standards for Bereavement Care aimed to provide recommendations from an expert group to ensure the delivery of compassionate care for perinatal loss [6]. Postgraduate training bodies then implemented educational initiatives aimed at supporting and preparing clinicians and midwives for this supportive role. The TEARDROP (Teaching, Excellent, pArent, perinatal, Deaths‐related, inteRactions, tO, Professionals) programme in Ireland—set up by a Pregnancy Loss Research Group at University College Cork [7]—is a 1‐day multidisciplinary workshop with interactive stations covering the core components of bereavement care. There is a focus on emphasising the lived experience and considered communication in bereavement care in the ‘Approach to Caring and Coping’ module, which was developed in a collaboration with the National Theatre of Ireland. This uses drama techniques to help trainee obstetricians to explore communication skills and their understanding of the complex emotions around pregnancy loss [8].

These programmes are designed to provide education to healthcare professionals who are *already* in clinical practice. Our project builds on this by introducing parent‐led bereavement education at the *student* level. At our institution, prior to the implementation of this educational initiative, there was no structured teaching programme including the voices of bereaved parents or directly addressing the emotional and personal aspects of pregnancy loss. Most students will encounter pregnancy loss in their future practice, so it is imperative that they have a baseline exposure to develop the competencies required to provide compassionate perinatal loss support [9, 10].

Clinicians often feel unprepared to provide support to grieving parents. This may be due to a lack of formal training, discomfort with bereavement conversations and difficulty in responding to the emotional needs of grieving parents [9]. Little emphasis is placed on grief training and communication skills around loss at an undergraduate level [11].

There is a recognition that core competencies for providing perinatal bereavement care extend beyond clinical knowledge. These include sensitive communication, self‐awareness and respect for parent autonomy and choices [6, 12]. This project is aimed at providing students with a unique opportunity to learn about bereavement care from those who are experts through lived experiences—parents themselves. Patient storytelling can promote student empathy, deepen understanding of personal experiences and support patient‐centred approaches to medical care.

The aim of this paper is to describe the development and implementation of a parent‐led educational initiative on perinatal bereavement and to evaluate its short‐term impact on students' self‐reported knowledge, skills and self‐awareness using a validated assessment tool for perinatal bereavement care.

*The aim of this paper is to describe the development and implementation of a parent‐led educational initiative on perinatal bereavement and to evaluate its short‐term impact on students' self‐reported knowledge, skills and self‐awareness using a validated assessment tool for perinatal bereavement care*.

## 2 Approach

### 2.1 Fitting Into the Current Curriculum

This educational initiative was developed for final‐year medical students at University College Dublin (UCD) taking part in a 6‐week Obstetrics and Gynaecology rotation. This module is designed to be an immersive and clinically focused module with students rotating through urban and rural placements. Education consists of didactic lectures, bedside tutorials, active participation in outpatient clinics and supervised exposure in the labour ward and operating theatres. Prior to this educational initiative, students received approximately 3 h of dedicated teaching on perinatal loss, including lectures on ectopic pregnancy, recurrent miscarriage, stillbirth and termination of pregnancy. There was a curricular gap identified at a local level with a lack of structured teaching focused on the emotional and personal aspects of pregnancy loss, and there were no included sessions highlighting the voices and perspectives of bereaved parents. An informal needs assessment, including discussions with module coordinators and students, highlighted a clear gap in bereavement education, including the fact that students reported no prior experience in bereavement care. This highlighted the necessity for early implementation on this important topic.

### 2.2 Design and Implementation of the Educational Sessions

This educational initiative consisted of three 1‐h sessions per 6‐week module. This was piloted and adapted based on suggestions from patient educators for the April–May 2024 and May–June 2024 student groups. The third cohort of 72 students (September–October 2024) participated in the evaluation described, which is now embedded into the module curriculum.

The educational sessions took place in one hospital classroom and were securely livestreamed to other clinical sites. Each session was designed to emphasise the personal narrative and storytelling of bereaved parents. The three advocacy groups involved included Féileacáin Ireland, LMC (Leanbh Mo Chroí/Child of my Heart) and Ectopic Pregnancy Ireland. All three groups represented different aspects of pregnancy loss and perinatal bereavement and are not‐for‐profit organisations (Table 1).

**TABLE 1:** Partner organisations involved in perinatal bereavement educational sessions and description of contribution made to each educational session.

Organisation	Focus of advocacy & support	Contribution to the session
Féileacáin (‘Butterfly’) ‐ Stillbirth and Neonatal Death Support	All forms of pregnancy loss	Speaker shared *personal experiences* of loss. Emphasised the importance of *memory making* rituals. Highlighted importance of *sensitive communication* and the involvement of the whole family.
Ectopic Pregnancy Ireland	Ectopic pregnancy	Highlighted the *emotional impact* of ectopic pregnancy. Emphasised the importance of *recognition* as ectopic pregnancy as a perinatal loss. Placed importance on awareness of the condition and *sensitive and considered communication*.
Leanbh Mo Chroí (‘Child of my Heart’)	Pregnancy and perinatal loss following antenatal diagnosis of a life‐limiting condition	Shared personal stories of loss secondary to termination of pregnancy for medical reasons. Focused on nonjudgemental and sensitive care. Highlighted the impact of recurrent pregnancy loss.

The educational sessions were planned and delivered by parents from the three organisations.

*Each session was designed to emphasise the personal narrative and storytelling of bereaved parents. The three advocacy groups involved included Féileacáin Ireland, LMC (Leanbh Mo Chroí/Child of my Heart) and Ectopic Pregnancy Ireland. All three groups represented different aspects of pregnancy loss and perinatal bereavement and are not‐for‐profit organisations*.

A variety of approaches were used with some choosing to use slides as visual aids and others opting for an entirely narrative approach. Students were encouraged by the parents to engage actively throughout the session by asking questions and getting involved in the discussion.

### 2.3 The Role of Parent‐Educators

This was a collaborative project between UCD and parent advocates from each of the named organisations. Parent‐educators were involved in the design and implementation of all educational sessions. Their feedback informed adjustments and changes to session structure. See Table 2 for details of the key areas of parent involvement.

**TABLE 2:** Summary of parent involvement and contributions to educational session implementation and delivery.

Focus of involvement	Description & examples
Session design	Parent‐educators made decisions on their preferred session format, e.g., stories delivered with the aid of slide presentations vs. purely narrative storytelling approach. Parents led decisions on room setup based on personal preferences, e.g., session delivered from lecture podium vs. seated in front of classroom.
Session delivery	Sessions led and facilitated by parent‐educator alone. Primary author (CK) introduced parent‐educator to class and remained for emotional/psychological support but did not interact during session.
Technical considerations	Camera placement modified based on parent‐educator feedback to enable interaction with remote students.
Student engagement	Parents suggested anonymous Q&A methods and presubmitted questions to enhance student engagement.
Language sensitivity	Parents recommended against using terms like ‘foetus’ when describing or introducing the sessions due to its potential to cause distress.
Postsession debrief and resources	Debrief between primary author (CK) and parent‐educators following session. Discussed challenges associated with each session and potential solutions for next iteration. Parents suggested learning materials, e.g., podcasts that can be included as additional learning resources for students. Parents provided students with contact information for support groups should they require confidential support following the sessions.

## 3 Evaluation

This was a pilot evaluation using a pre–post design to explore the immediate self‐reported impact of the programme on predefined areas of bereavement knowledge, skills and self‐awareness. The Perinatal Bereavement Care Confidence Scale (PBCCS) was used as an evaluation tool [13]. This is a scoring system that was originally designed to evaluate midwives and nurses' confidence levels in caring for bereaved parents and has been used in a mixed population of healthcare providers since [14]. Three separate domains explore bereavement knowledge, bereavement support skills, self‐awareness and organisational support, with a short area for free‐text responses. See Table 3 for free text response options.

**TABLE 3:** Free text comments included in Perinatal Bereavement Care Confidence Scale.

Comment 1	Comment 2	Comment 3
What other things promote your confidence to provide support to bereaved parents?	What other things inhibit your confidence to provide support to bereaved parents?	If you have other suggestions on how your confidence for providing bereavement support to grieving parents could be promoted, please write them below

The authors of the PBCCS were contacted in advance of the educator sessions to obtain permission and instructions on its correct use. The domain on organisational support was omitted due to its lack of relevance to the student participants, as was a question on specific reflective practice. A copy of the modified PBCCS can be found in Appendix S1.

### 3.1 Learning Outcomes

The intended learning outcomes for the educational initiative aligned directly with the core domains for the PBCCS and can be seen outlined in Table 4.

**TABLE 4:** Intended learning outcomes directly aligned with the core domains of the PBCCS.

Learning outcomes	PBCCS domain	Sample PBCCS items
Recognise the emotional impact of perinatal loss on parents	Knowledge	2.1 ‘Perinatal loss is a traumatic event for bereaved parents’ 2.3 ‘I understand that grieving is a process’
Understand the importance of sensitive communication	Skills	3.4 ‘I can comfortably listen to bereavement parents without trying to interrupt them’
Develop greater empathy through hearing about lived experiences	Self‐awareness	4.5 ‘I am aware of the needs of recently bereaved parents’

### 3.2 Data Collection

Data collection for this study was as follows:

1. Paper copies of the PBCCS were distributed in‐person to all 72 students at the induction session on their first day of the module. Students were given the opportunity to complete the forms anonymously and return them via a confidential submission box on the same day.
2. Immediately following the completion of the third and final patient educator session, paper copies of the questionnaire were distributed at each clinical site, and students were requested to place any completed forms into the submissions box.

### 3.3 Data Analysis

Demographic data including student gender, age range and educational level were analysed in Microsoft Excel using descriptive statistics. Quantitative data from the PBCCS were analysed using IBM SPSS Statistics Version 29.0.2. Responses were grouped into the three core domains (knowledge, skills and self‐awareness) and analysed as composite scores. To assess normality, skewness and kurtosis values were calculated for the composite scores in each domain. The knowledge and skills domains met criteria for normality and were analysed using independent samples *t* tests. The self‐awareness domain did not strictly meet the criteria for parametric testing (Kurtosis 1.42); the Mann–Whitney *U* test was used for this domain. Free text responses were analysed using thematic content analysis to identify recurring themes related to confidence‐promoters, confidence‐inhibitors and student suggestions for improving bereavement education.

### 3.4 Ethical Approval

Ethical approval for this study was granted by UCD Research Ethics Committee in April 2024 (LS‐C‐24‐19‐Kennedy–Higgins).

## 4 Results

Seventy‐two students attended the educational programme. Of these students, 60 completed the presession PBCCS, and 35 completed the postsession PBCCS. As students completed the PBCCS anonymously and without identifiers, the groups were treated as independent rather than paired samples. The gender distribution was identical across both groups (60% female and 40% male). Most (> 88%) students were aged between 20 and 29 years, and most (98.3%) reported no previous experience in bereavement care.

### 4.1 Quantitative Findings From the PBCCS

**FIGURE 1:**
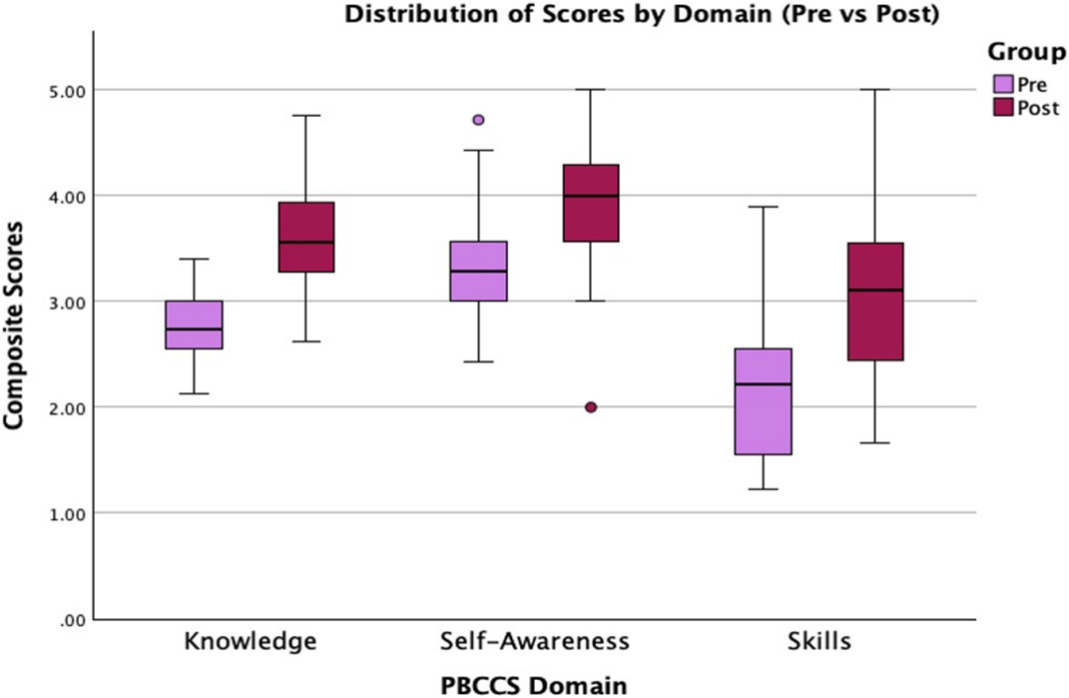
Distribution of scores across each domain before and after the educational intervention.

Statistically significant improvements were observed across all three domains of the PBCCS following the educational sessions (see Figure 1). Knowledge scores increased from a mean of 2.80–3.59 (*p* < 0.01; Cohen's *d* = 0.39), indicating a small to medium effect. Skills scores rose from a mean of 2.16–3.09 (*p* < 0.001; *d* = 0.65), indicating a medium effect. Self‐awareness scores increased from a median of 3.29–4.0 (< 0.001).

### 4.2 Qualitative Findings From Free‐Text Responses

The students' responses to the open‐ended questions offered additional insight into what shaped their confidence in providing bereavement care, as well as the areas where further support is needed. Before the sessions, students consistently reported ‘limited experience’ as a barrier, for example, also stating ‘I have no education on this matter’. Many also described worry or anxiety about making mistakes with one stating they were ‘scared of saying the wrong thing’.

*Before the sessions, students consistently reported ‘limited experience’ as a barrier, stating ‘I have no education on this matter’. Many also described worry or anxiety about making mistakes, with one stating they were ‘scared of saying the wrong thing’*.

Promoters of confidence prior to the sessions were sparse; however, some students highlighted the potential value in learning from patients stating ‘exposure … where parents tell their story’ as a possible confidence promoter. Following the session, many students described the impact of hearing directly from bereaved parents. One student noted, ‘The patient education sessions that we've had in this module were very informative and thought provoking. Attending more of these session will give me confidence to provide support’. They also described the valuable information gained from the sessions including ‘A new knowledge of some of the disorders which may result in perinatal loss’. Suggestions for further improvement included ‘More teaching on how to talk to a patient’ and ‘continuing educator sessions’.

## 5 Implications

This pilot demonstrates that parent‐led educational initiatives based on a story‐telling approach can improve final‐year medical students' self‐reported bereavement knowledge, skills and self‐awareness surrounding bereavement care. These improvements are further supported by free‐text responses, with students highlighting how hearing from bereaved parents helped them to develop insight into the lived experience of pregnancy loss. This aligns with previous research showing the impact of patient narratives on student empathy levels [15]. This programme addresses a gap in the current curriculum by incorporating the patient voice and lived experience, giving students a new perspective that cannot be obtained with didactic teaching alone. The study demonstrates that targeted, patient‐led educational interventions can have a meaningful impact on students' bereavement skills and knowledge and therefore has the potential to improve care.

*This pilot demonstrates that parent‐led educational initiatives based on a story‐telling approach can improve final year medical students' self‐reported bereavement knowledge, skills and self‐awareness surrounding bereavement care*.

### 5.1 Future Directions

This educational initiative has now been formally integrated into the Obstetrics and Gynaecology module in UCD. Parent‐educators are offered remuneration for their time; this is integral to the sustainability of the programme and acknowledges their expertise. Ongoing improvements to the programme are guided by discussions with parent‐educators. Feedback has already led to refinements in several areas, including earlier scheduling of sessions within the rotation to optimise student engagement or implementation of suggestions to improve participation (e.g., offering students the opportunity to submit questions in advance or contribute anonymously during the sessions through online platforms). This educational initiative adds to the growing body of evidence supporting patient involvement in medical education. Further research may include longitudinal follow‐up to better understand the long‐lasting impact of this type of educational programme.

### 5.2 Limitations

As a pilot initiative, a preintervention and postintervention survey design was used with nonpaired anonymous responses, which limits individual comparisons between students. Response rates at the postintervention survey were lower, which may introduce bias. Although composite scores for each domain in the PBCCS were analysed, future research will include a more detailed analysis of the individual components of the survey.

## Author Contributions

**Clare Kennedy:** conceptualization, formal analysis, investigation, methodology, project administration, resources, visualization, writing – original draft. **Alison Lynch:** methodology, writing – review and editing. **Nina Doyle:** methodology, writing – review and editing. **Susanne Brodigan:** methodology, writing – review and editing. **Laura Guild:** methodology, writing – review and editing. **Mary F. Higgins:** conceptualization, methodology, supervision, writing – review and editing, project administration.

## Ethics Statement

Ethical approval to carry out this study has been granted by the Research Ethics Committee, University College Dublin.

## Conflicts of Interest

The authors declare no conflicts of interest.

## Supporting information


**Appendix S1** Supporting Information.

## Data Availability

The data that support the findings of this study are available from the corresponding author upon reasonable request.
