# Supplementary material for: ‘Stories of Loss’—Designing and Evaluating a Patient‐Led Perinatal Bereavement Programme for Medical Students
Source: Clin Teach. 2025 Jul 16;22(4):e70146. doi: 10.1111/tct.70146 (PMC12268117; doi:10.1111/tct.70146)
Supplement: Supplementary file 1 — Appendix S1 Supporting Information. [file TCT-22-e70146-s001.docx]

**Perinatal Bereavement Care Confidence Scale (PBCCS)**

This is an anonymous survey, and you are **not** asked to sign your name. By voluntarily completing and returning the questionnaire, you are consenting to participate in this research.

**Section 1**

**Demographic details**

This section contains questions that will provide information about your background. Please tick the appropriate boxes for the following questions

1. What is your gender?

Male Female Choose not to answer

1. What is your age in years?

20-29 30-39 40- 49 50-59 60 or above

1. What is your highest level of education?

Certificate Diploma Higher Diploma Postgraduate Diploma

Bachelor’s degree Master’s degree Doctorate Leaving Certificate (Please specify)

1. Have you received any perinatal bereavement support education?

Yes No

If yes, was it through: formal academic programme, study day, OR Other (Please specify)

_____________________________________________________________________

**Section 2**

**Perinatal bereavement knowledge**

Listed below are a number of statements. Please read each statement and tick one answer in the box that describes how much you agree with the statement right now.

|  | Statements | 1  Strongly Disagree | 2  Disagree | 3  Neither Agree/  Disagree | 4  Agree | 5  Strongly Agree | |
| --- | --- | --- | --- | --- | --- | --- | --- |
| 2.1 | Perinatal loss is a traumatic event for bereaved parents |  |  |  |  |  | |
| 2.2 | Bereaved parents require the support of clinical staff to cope with their loss |  |  |  |  |  | |
| 2.3 | I understand that grieving is a process |  |  |  |  |  | |
| 2.4 | I know how to provide the specific bereavement support needs of grieving mothers |  |  |  |  |  | |
| 2.5 | I understand the cultural needs of bereaved parents |  |  |  |  |  | |
| 2.6 | I understand the social needs of bereaved parents |  |  |  |  |  | |
| 2.7 | I do not know the legal process associated with perinatal loss before 24 weeks gestation |  |  |  |  |  | |
| 2.8 | I do not know how to provide the specific bereavement support needs of grieving fathers |  |  |  |  |  | |
| 2.9 | I understand the religious needs of bereaved parents |  |  |  |  |  | |
| 2.10 | I know the referral system for additional bereavement support |  |  |  |  |  | |
| 2.11 | I do not have adequate practical knowledge for bereavement support |  |  |  |  |  | |
| 2.12 | I know the legal process associated with perinatal loss after 24 weeks gestation |  |  |  |  |  | |
| 2.13 | I have been well prepared to provide perinatal bereavement support |  |  |  |  |  | |
| 2.14 | There is a need for continuing perinatal bereavement education for maternity care professionals. |  |  |  |  |  | |
| 2.15 | All maternity care professionals at the hospital should receive perinatal bereavement education |  |  |  |  |  |  |

**Section 3**

**Skills for providing perinatal bereavement support**

Please tick one answer in the box that describes your level of agreement with each of the following statements

|  | Statements | 1  Strongly Disagree | 2  Disagree | 3  Neither Agree/  Disagree | 4  Agree | 5  Strongly Agree |
| --- | --- | --- | --- | --- | --- | --- |
| 3.1 | I have the skills to provide practical support to recently bereaved parents |  |  |  |  |  |
| 3.2 | I do not have adequate perinatal bereavement support experience |  |  |  |  |  |
| 3.3 | I have grief counselling skills for providing psychological support to bereaved parents |  |  |  |  |  |
| 3.4 | I can provide the relevant information required by bereaved parents |  |  |  |  |  |
| 3.5 | I can comfortably listen to bereaved parents without trying to interrupt them. |  |  |  |  |  |
| 3.6 | I can provide emotional care to bereaved parents |  |  |  |  |  |
| 3.7 | I can provide spiritual care to bereaved parents |  |  |  |  |  |
| 3.8 | I can easily respond to the needs of bereaved sibling when accompanying their parents |  |  |  |  |  |
| 3.9 | I can easily respond to the needs of bereaved parents expecting their next baby |  |  |  |  |  |

**Section 4**

**Self-awareness**

Please tick one answer in the box that describes your level of agreement with each of the following statements

|  | Statements | 1  Strongly Disagree | 2  Disagree | 3  Neither Agree/  Disagree | 4  Agree | 5  Strongly Agree |
| --- | --- | --- | --- | --- | --- | --- |
| 4.1 | I am aware of the needs of recently bereaved parents |  |  |  |  |  |
| 4.2 | I can easily empathise with grieving parents (Empathy means that I can emotionally put myself in their place). |  |  |  |  |  |
| 4.3 | I am conscious of the particular needs of bereaved parents expecting their next baby |  |  |  |  |  |
| 4.4 | I am aware of my limitations in relation to the provision of perinatal bereavement support |  |  |  |  |  |
| 4.5 | I am aware of my learning needs regarding bereavement support |  |  |  |  |  |
| 4.7 | I am aware of my personal resources for bereavement support |  |  |  |  |  |
| 4.8 | Being aware of my need for support in relation to providing care for bereaved parents encourages me to seek help |  |  |  |  |  |

What other things promote your confidence to provide support to bereaved parents?

___________________________________________________________________________

______________________________________________________________________________________________________________________________________________________

What other things inhibit your confidence to provide support to bereaved parents?

_________________________________________________________________________________________________________________________________________________________________________________________________________________________________

If you have other suggestions on how your confidence for providing bereavement support to grieving parents could be promoted, please write them below.

_________________________________________________________________________________________________________________________________________________________________________________________________________________________________

Thank you for taking part in this study.
